# Advancements and Perspectives in Nanotechnology and Nanomedicine

**DOI:** 10.3390/v17050638

**Published:** 2025-04-29

**Authors:** Olga Morozova

**Affiliations:** 1 Institute of Future Biophysics, 9/7 Institutsky Per., Dolgoprudny 141700, Moscow Region, Russia; omorozova2010@gmail.com; 2 Federal Research and Clinical Center of Physical-Chemical Medicine of Federal Medical Biological Agency, 1a Malaya Pirogovskaya Street, Moscow 119435, Russia; 3 Ivanovsky Institute of Virology of the National Research Center of Epidemiology and Microbiology of N.F. Gamaleya of the Russian Ministry of Health, 16 Gamaleya Street, Moscow 123098, Russia

Progress in atom visualization and miniaturization in microelectronics resulted in the discovery of nanomaterials in the second half of the 20th century. Currently, nanotechnology is one of the driving forces in the development of technical and medical systems. Due to their high surface-to-volume ratios, nanomaterials possess novel physical, chemical and biological properties, and offer unique advantages for vaccines, diagnostic systems and the treatment of infections, cancer and conformational neurodegenerative diseases. Nanostructures are well known for their ability to permeate tissues, to accumulate inside the cells and effectively release the payloads into the cells, to enhance pharmacokinetic stability and to improve bioavailability, as well as for their biological barrier-crossing capacities, distribution among organs and modulation of both innate and adaptive immune response. Because of their enhanced permeability and retention (EPR) in cancer cells and intracellular drug delivery capabilities, nanomaterials are utilized in tumor therapy.

The number of nanotherapies approved by the US Food and Drug Administration [1] and the European Medicines Agency [2] remains limited due to possible toxicity and non-specific cellular uptake resulting in off-target effects. In addition, nanomedicines possess inherent immunomodulatory properties that can cause side effects [3].

Viruses can penetrate permissive cells and do not accumulate in the extracellular matrix and fluids for a long time. Therefore, artificial nonpathogenic virus-like particles can be used for drug targeting into specific cell populations. The Special Issue “Viruses and Virus-Like Particles as Nanoplatforms for Vaccines, Diagnostic and Therapeutic Nanomedicine” includes articles devoted to self-assembling nanoparticles from recombinant proteins of different origin. Despite the Special Issue being closed, the precise chemical structures with amino acid substitutions, the detailed electron and atomic force microscopic analysis of the virus-like particles with high resolution, along with their immunogenic profiles remain basic research for further implementation.

However, intracellular distribution and biodegradation of nanomaterials, including virus-like particles, are the main concerns for their biomedical and subsequent clinical applications. For polar and charged biopolymers, as well as for corresponding nanomaterials, passive diffusion through hydrophobic cellular membranes is hardly possible. Cellular uptake relies on four known mechanisms: endocytosis, phagocytosis, macropinocytosis and pinocytosis [4]. In the absence of specific receptors, clathrin/caveolar-independent endocytosis prevails. The internalization of nanostructures is based on the endocytic pathway, through which the particles remain trapped in endosomes and lysosomes [4], where more than 60 lysosomal enzymes catalyze the biodegradation of proteins, nucleic acids, carbohydrates and lipids. The enzymes require an acidic environment with the lumen’s pH ~4.5–5.0 for optimal binding and catalytic activity. Therefore, regardless of the protein and cell type, protein nanoparticles in cells are eliminated by 5 days post-treatment [5]. The proteolysis leads to the formation of short peptides and antigen presentation with subsequent expression of interferon genes and the T-helper type 1 (Th1) immune response [5]. Upon mucosal administration of protein nanoparticles to laboratory animals, their accumulation is observed in various parts of the brain, including the olfactory bulbs, the cerebellum and the cerebral cortex, as well as in the intestine, with peaks at 1–2 days post-treatment [6].

To escape from endosomal/lysosomal entrapment with accelerated biodegradation, an alternative entry is required. Envelope viruses enter cells via membrane fusion, that is mediated by the fusogenic virus surface glycoprotein oligomer complex [7,8]. In fusion, the virus membrane becomes contiguous with the cell membrane, whereas during endocytosis, the host cells internalize the virus by wrapping it in an endosomal vesicle. Composite nanoparticles consisting of a protein core and membrane shells can undergo both membrane fusion and endocytosis, with gradual accumulation inside cells over 7 days accompanied by low levels of cytokine response (if any) [9].

Amyloidogenic proteins derived from both RNA- and DNA-containing viruses with single-stranded and double-stranded genomic nucleic acids as well as from cells can cause severely impaired health [10,11]. The E7 protein of human papillomavirus (HPV)-16, the influenza A virus accessory protein PB1-F2 [10], and the self-assembling nanostructures derived from the SARS-CoV-2 S1, S2, and N recombinant proteins isolated from the transformed bacterial *Escherichia coli* cells, as well as the RBD fragment of the glycoprotein S produced in the transfected eukaryotic cells can form amyloid-like fibers and nanoparticles [11]. Self-assembling virus-like nanoparticles can hamper quantitative diagnostics and immunization with probable conformational proteinopathies. Therefore, all novel nanomaterials including non-toxic virus-like particles must be evaluated for their potential to contribute to proteinopathies.
